# Automated analysis of heart sound signals in screening for structural heart disease in children

**DOI:** 10.1007/s00431-024-05773-3

**Published:** 2024-09-21

**Authors:** I. Papunen, K. Ylänen, O. Lundqvist, M. Porkholm, O. Rahkonen, M. Mecklin, A. Eerola, M. Kallio, A. Arola, J. Niemelä, I. Jaakkola, T. Poutanen

**Affiliations:** 1 Tampere Center for Child, Adolescent and Maternal Health Research, Faculty of Medicine and Health Technology, Tampere University, Tampere, Finland; 2 Department of Pediatrics, Tampere University Hospital, Wellbeing Services County of Pirkanmaa, Tampere, Finland; 3 AusculThing Oy, Espoo, Finland; 4 Department of Pediatric Cardiology, New Children’s Hospital, University of Helsinki and Helsinki University Hospital, Helsinki, Finland; 5 Department of Pediatrics and Adolescent Medicine, Oulu University Hospital and University of Oulu, Oulu, Finland; 6 Department of Pediatrics and Adolescent Medicine, Turku, Finland

**Keywords:** Artificial intelligence, Congenital heart defect, Electronic stethoscope, Heart murmur, Screening

## Abstract

Our aim was to investigate the ability of an artificial intelligence (AI)-based algorithm to differentiate innocent murmurs from pathologic ones. An AI-based algorithm was developed using heart sound recordings collected from 1413 patients at the five university hospitals in Finland. The corresponding heart condition was verified using echocardiography. In the second phase of the study, patients referred to Helsinki New Children’s Hospital due to a heart murmur were prospectively assessed with the algorithm, and then the results were compared with echocardiography findings. Ninety-eight children were included in this prospective study. The algorithm classified 72 (73%) of the heart sounds as normal and 26 (27%) as abnormal. Echocardiography was normal in 63 (64%) children and abnormal in 35 (36%). The algorithm recognized abnormal heart sounds in 24 of 35 children with abnormal echocardiography and normal heart sounds with normal echocardiography in 61 of 63 children. When the murmur was audible, the sensitivity and specificity of the algorithm were 83% (24/29) (confidence interval (CI) 64–94%) and 97% (59/61) (CI 89–100%), respectively.

*Conclusion*: The algorithm was able to distinguish murmurs associated with structural cardiac anomalies from innocent murmurs with good sensitivity and specificity. The algorithm was unable to identify heart defects that did not cause a murmur. Further research is needed on the use of the algorithm in screening for heart murmurs in primary health care.

**Table:** 

What is Known: *• Innocent murmurs are common in children, while the incidence of moderate or severe congenital heart defects is low. Auscultation plays a significant role in assessing the need for further examinations of the murmur. The ability to differentiate innocent murmurs from those related to congenital heart defects requires clinical experience on the part of general practitioners. No AI-based auscultation algorithms have been systematically implemented in primary health care.*	
What is New: *• We developed an AI-based algorithm using a large dataset of sound samples validated by echocardiography. The algorithm performed well in recognizing pathological and innocent murmurs in children from different age groups.*

What is Known:

*• Innocent murmurs are common in children, while the incidence of moderate or severe congenital heart defects is low. Auscultation plays a significant role in assessing the need for further examinations of the murmur. The ability to differentiate innocent murmurs from those related to congenital heart defects requires clinical experience on the part of general practitioners. No AI-based auscultation algorithms have been systematically implemented in primary health care.*

What is New:

*• We developed an AI-based algorithm using a large dataset of sound samples validated by echocardiography. The algorithm performed well in recognizing pathological and innocent murmurs in children from different age groups.*

## Introduction

A heart murmur can be a sign of a congenital heart defect (CHD), but innocent murmurs are common in children and can be heard in 50% of school-aged children [1, 2]. CHDs are found in about 75 per 1000 live-born infants. However, the incidence of moderate or severe CHD is only six cases per 1000 live births [3]. About 10% of children with significant CHDs are discharged from the maternity hospital without receiving a diagnosis [4], and CHDs are rarely found in children over 6 months of age [5, 6]. Although a murmur is the most common finding that raises suspicion of CHD, most of the children referred to pediatric cardiologists have an innocent murmur. According to some estimates, up to 90% of initial visits to pediatric cardiology clinics were due to an innocent murmur and thus unnecessary [5, 7].

Despite the development of medical technology, auscultation still plays a significant role in assessing the need for further examinations of the murmur. The interpretation of cardiac auscultation is subject to uncertainty because it depends on the subjective perceptions and experience of the clinician. Computer decision algorithms based on artificial intelligence (AI) have been developed in recent years to identify murmurs collected by electronic stethoscopes [8]. The sensitivity and specificity of an AI-assisted murmur detection algorithm were shown to be good in differentiating innocent murmurs from pathologic ones in a validation study involving both adults and children [9]. To the best of our knowledge, only a few studies have been published on pediatric populations, with the algorithms differentiating pathologic murmurs from normal heart sounds with good sensitivity [9, 10]. While the use of AI has potential for cost-effective CHD screening, it has not yet been utilized in clinical practice. Our aim was to evaluate the ability of an AI-based algorithm to identify innocent and pathologic murmurs in children.

## Materials and methods

### The development of the algorithm

An AI-based algorithm was developed by fine-tuning a state-of-the-art speech model using heart sound recordings collected at five university hospitals in Finland. The heart sound recordings, selected for the training of the algorithm, were collected from 1413 patients, including 1061 (75%) children (< 18 years) and 352 (25%) adults (Table 1).

**Table 1:** Basic information of the patients used to train the algorithm (*N* = 1413)

**Characteristic**	*N* = 1413 (%)
**Sex** Female	671 (47%)
**Age** 0–1 month 1–6 months 6–12 months 1–4 years 4–12 years 12–18 years > 18 years	83 (6%) 155 (11%) 66 (5%) 143 (10%) 375 (27%) 239 (17%) 352 (25%)
**Heart murmur** No murmur and no CHD Innocent murmur Pathological murmur	514 (36%) 339 (24%) 560 (40%)
**Result of echocardiography** Normal Abnormal	853 (60%) 560 (40%)

**Fig. 1:**
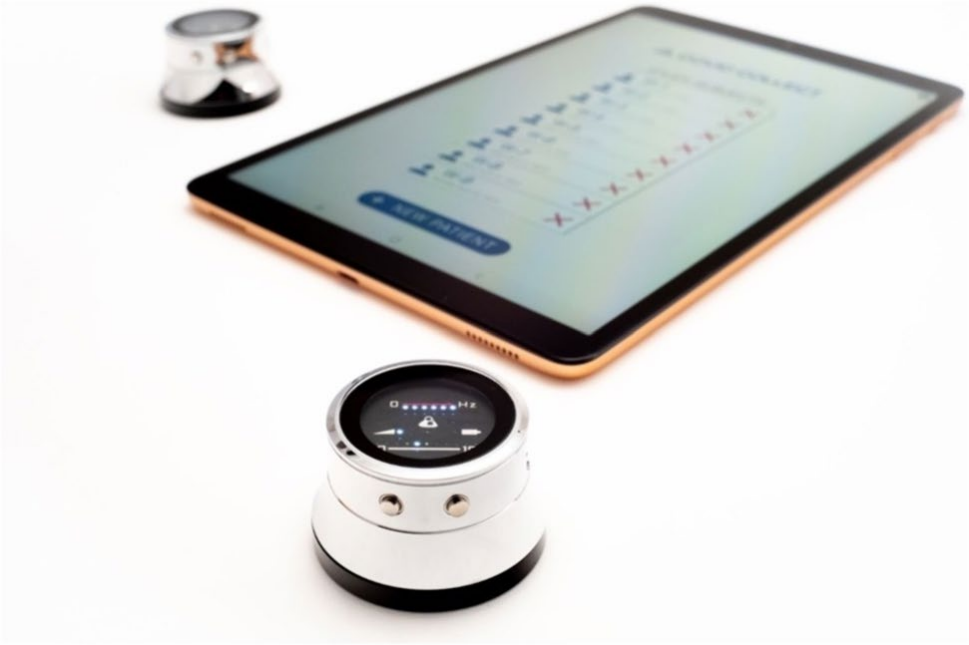
Equipment for analysis of the heart sounds

The recordings from CHDs without murmurs were not used in the development of the algorithm. The recordings were made with the Thinklabs One® electronic stethoscope, which is commercially available and is licensed for medical use by the manufacturer (US FDA Class 2 diagnostic device 2004 and CE-marking 2017). The data were collected and saved on a Samsung Galaxy Tab A10 tablet (CE) (Fig. 1). Recordings were made from four different locations (aortic, pulmonary, tricuspid, and mitral valve areas), with a sample rate of 44 kHz and a duration of 10 s. If patient co-operation was not adequate to complete all four recordings with good signal quality, only the samples with adequate signal were included in training the algorithm.

**Fig. 2:**
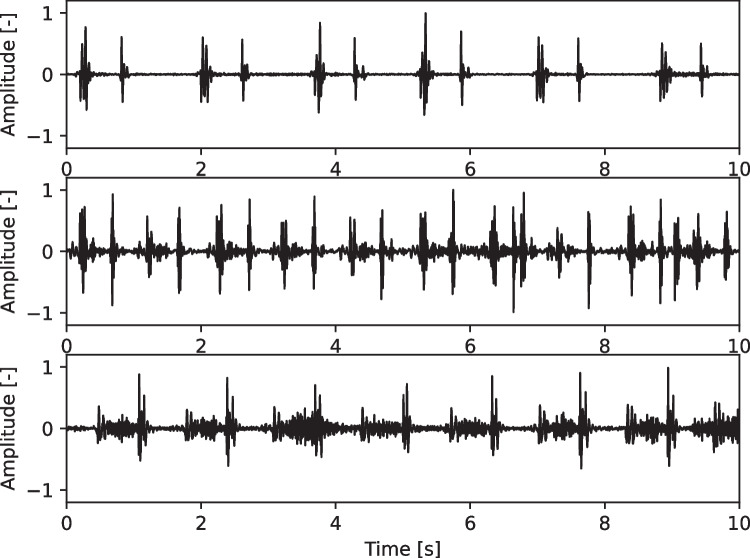
Phonocardiograms of normal heart sounds (top), innocent murmur (middle), and ventricular septal defect (bottom). This recording is the technical input to the AI algorithm

The trained algorithm was divided into two leading AI algorithms. The quality component is a neural network trained to extract high-quality data from the raw recordings by masking noise such as screaming/stethoscope movements. The high-quality components from the raw recordings are passed to the binary classifier which is used to detect abnormalities in the recording. A binary classifier inputs the high-quality phonocardiogram segments and used this information to predict the presence or absence of a heart defect causing an audible heart murmur (Fig. 2). The algorithm formed a floating-point value between 0 and 1. A threshold of 0.5 was set as the divider for the two categories of normal and abnormal heart sounds. If the output value (probability of defect) was less than 0.5, the result was classified as normal, and an output value above 0.5 was classified as abnormal. The higher/lower the number the more confident the model is in its prediction—the closer the output is to the threshold of 0.5 and the more uncertainty is related to the prediction. The normal category included heart sounds with no murmur and innocent murmurs, while the abnormal heart sound included pathological murmurs. Both algorithms were trained using the same training dataset and utilized the same separate development set for hyperparameter tuning and establishing performance metrics on data not used during training to understand how well each model generalized on unseen data. The development set was constructed using a 90–10 split (90% of the data was used for training, and 10% was placed in the development set). The development set was not the same dataset as the result set of the 98 patients, which was used in this research.

### Study patients

A convenience sampling method consisting of 98 children was used in the study. Patients were prospectively recruited during their outpatient visits at the Pediatric Cardiology unit at the New Children’s Hospital in Helsinki, Finland, between Mar 29, 2022 and Aug 9, 2023. The patients were examined by a pediatric cardiologist due to a heart murmur or CHD. Children with previous heart surgery or insufficient co-operation were excluded. The patients were first analyzed with the algorithm, and then the results were compared with clinical evaluations and echocardiography. The pediatric cardiologists performed auscultation with a conventional stethoscope before the echocardiography and assessed whether a murmur was audible. The cardiologist was not aware of the analysis result of the AI. Finally, echocardiography was performed, and the findings were classified as normal or abnormal. The algorithm’s analysis of the murmur (normal or abnormal) was compared with the diagnosis made by echocardiography. The algorithm result “abnormal” included all pathologic murmurs identified by the algorithm. The result “normal” included innocent murmurs and normal heart sounds without murmurs.

All the study patients and/or their legal guardians provided written informed consent. This study complied with the Declaration of Helsinki and was approved by the ethical committee (HUS/1630/2019 August 23, 2019) of Helsinki University Hospital (HUS).

The statistical analyses were performed using IBM SPSS Statistics for Macintosh (version 29.0.1.0). Categorical values were expressed as frequencies and percentages. The median and interquartile range (IQR) (Q1–Q3) were expressed for the non-normally distributed variables. The sensitivity, specificity, and accuracy of the algorithm to differentiate an innocent murmur from a pathologic one were calculated. Categorical variables were compared with chi-square tests. Sensitivity, specificity, and accuracy were calculated with 95% confidence intervals.

## Results

### Study population

Heart sounds of 98 pediatric patients were analyzed in this study. The patient characteristics and echocardiography findings are presented in Table 2.

**Table 2:** Basic patient data of the patients examined with the algorithm

Characteristic	*N* = 98 (%)
**Sex** Female	50 (51%)
**Age** 0–1 month 1–6 months 6–12 months 1–4 years 4–12 years > 12 years	3 (3%) 14 (14%) 7 (7%) 20 (20%) 31 (32%) 23 (24%)
**Heart murmur** Yes	90 (92%)
**Result of echocardiography** Normal Abnormal* Shunt lesions Valvular lesions Other lesions	63 (64%) 35 (36%) 22 (22%) 17 (17%) 3 (3%)

*One patient can have more than one diagnosis

Echocardiography was normal in 63 (64%) children and abnormal in 35 (36%).

The algorithm identified 72 (73%) of the heart sounds as normal and 26 (27%) as abnormal. The sensitivity of the algorithm was 69% (CI 51–83%), and the specificity was 97% (CI 89–100%) (Table 3).

**Table 3:** Performance of the AI-based algorithm in detecting pathologic murmurs verified by echocardiography. All patients (*N* = 98)

		Echocardiography		
		Abnormal	Normal	
Algorithm	Abnormal	True positive 24	False positive 2	Positive predictive value 0.92
	Normal	False negative 11	True negative 61	Negative predictive value 0.85
		Sensitivity 0.69	Specificity 0.97	Accuracy 0.87

The algorithm recognized pathologic murmurs in 24 of 35 cases with abnormal echocardiography and misdiagnosed 11 cases. Of the 11 misdiagnosed cases, five had an audible murmur and six had no murmur. The algorithm did not identify murmurs caused by aortic valve insufficiency (diastolic grade I), patent ductus arteriosus (PDA) (grade 1 systo-diastolic), atrial septal defect (ASD), or a small ventricular septal defect (VSD). In total, 61 of 63 children with normal echocardiography findings were identified as having normal heart sounds by the algorithm. The level of confidence of the algorithm was unsure in six (6%) analyses and confident in 92 (94%). The details of the algorithm analyses and the findings of the patients with abnormal echocardiography are presented in Table 4. In the group of patients with abnormal echocardiography findings, the median (IQR) probability set by the algorithm of a CHD was 0.66 (0.27–0.98) (Table 4).

**Table 4:** The diagnoses found in the echocardiography (*n* = 35) classified according to the interpretation of the algorithm

Algorithm analysis	Diagnoses	Murmur	*n*	Probability of defect (range)
Abnormal *n* = 24	Ventricular septal defect Atrial septal defect Aortic stenosis Pulmonary stenosis Coarctation of aorta Patent ductus arteriosus Aortic and pulmonary stenosis Others	Yes Yes Yes Yes Yes Yes Yes Yes	7 4 4 3 2 1 1 2	0.54–0.99 0.58–0.99 0.87–0.97 0.99 0.78–0.87 0.92 0.98 0.94–0.95
Normal *n* = 11	Bicuspid aortic valve Ventricular septal defect Atrial septal defect Atrial septal defect Ventricular septal defect Aortic valve insufficiency Patent ductus arteriosus	No No No Yes Yes Yes Yes	2 2 2 2 1 1 1	0.03 0.09–0.25 0.18–0.33 0.11–0.49 0.41 0.04 0.07

When comparing age groups, the algorithm had a sensitivity of 75% (12/16) (CI 48–93%) and a specificity of 96% (27/28) (CI 82–100%) in the group of children aged 0–4 years (*n* = 44). In children over 4 years of age (*n* = 54), the sensitivity was 63% (12/19) (CI 38–84%), while the specificity was 97% (34/35) (CI 85–100%). There was one CHD without a murmur in children aged 0–4 years (VSD) and five in children over 4 years of age (VSD, bicuspid aortic valve, and ASD) (Table 4).

### Patients with a murmur

Pediatric cardiologists heard a murmur from 90 of 98 patients (92%). In these 90 patients, the sensitivity of the algorithm was 83% (24/29) (CI 64–94%) and the specificity was 97% (59/61) (CI 89–100%) (Table 5). The algorithm was confident in 83 (93%) and unsure in six (7%) analyses.

**Table 5:** Performance of the AI-based algorithm in detecting pathologic murmurs verified by echocardiography among patients with audible murmur (*N* = 90)

		Echocardiography		
		Abnormal	Normal	
Algorithm	Abnormal	True positive 24	False positive 2	Positive predictive value 0.92
	Normal	False negative 5	True negative 59	Negative predictive value 0.94
		Sensitivity 0.86	Specificity 0.97	Accuracy 0.93

**Fig. 3:**
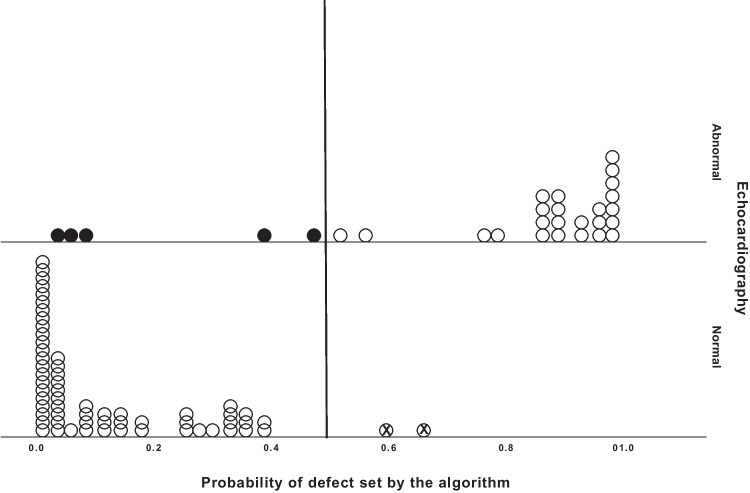
The probability of congenital heart defect as analyzed by the algorithm compared to echocardiography results in the patients with a murmur (*n* = 90)

The median (IQR) probability of CHD was 0.15 (0.03–0.27) in children with normal echocardiography findings and an audible murmur and 0.81 (0.79–0.99) in children with abnormal echocardiography findings and an audible murmur (Fig. 3). Comparing age groups, the sensitivity of the algorithm was 80% (12/15) (CI 52–96%) and its specificity was 96% (26/27) (CI 81–100%) in the group of children aged 0–4 years (*n* = 42). In children over 4 years of age (*n* = 48), the algorithm’s sensitivity was 86% (12/14, CI 57–98%) and its specificity was 97% (33/34, CI 85–100%).

The vertical black line marks the threshold probability of the defect (0.5). The black dots represent the abnormalities that were not detected by the algorithm. The dots with “X” are the normal cases interpreted incorrectly as abnormal. The white dots represent the cases that the algorithm identified correctly.

## Discussion

This is the first clinical study to investigate the ability of AI to differentiate between benign and pathological heart murmurs in children to this extent using echocardiography as the gold standard. The algorithm was able to distinguish a murmur caused by a CHD from innocent murmurs with good sensitivity and specificity when echocardiography was used as the reference. Since the method is based on the identification of different heart sound signals, it is not suitable for screening CHDs without a murmur.

Despite the routine use of pulse oximetry screening, which is highly sensitive in detecting critical cyanotic CHDs, some newborns with acyanotic CHDs may still go undiagnosed before discharge from the maternity ward [11]. Indeed, many CHDs do not cause symptoms during the first week of life [4], and some are only diagnosed after normal postnatal adaptation has taken place. For example, VSD causes murmur after a reduction in pulmonary vascular resistance leads to a pressure difference between ventricles and coarctation when the arterial duct has closed and caused narrowing of the aorta. CHDs diagnosed in children over 6 months of age are usually mild and asymptomatic, including ASD, PDA, and valvular defects [6]. Some valvular defects and hypertrophic cardiomyopathy may manifest later in adolescence. Auscultation during childhood plays a vital role in identifying these CHDs, but the accuracy of interpretation depends on the experience and skills of the physician. In previous studies, the sensitivity and specificity of clinical assessments of CHD have varied widely depending on the clinical experience of the physician, while specificity generally increased with experience. For example, medical students and pediatric residents had a sensitivity of 82% but a specificity of only 56% in assessing CHD [12]. Among pediatricians, sensitivity was found to be better (93%), but specificity remained low (59%) [6]. Meanwhile, clinical assessment by pediatric cardiologists had a sensitivity of 81% and specificity of 91% in identifying neonates with CHD [13].

Auscultation using a conventional stethoscope or AI requires optimal conditions, including good patient co-operation and quiet environment. If a child is crying, both the physician and the algorithm may struggle to recognize murmurs accurately. In our study, all children were co-operative, ensuring a reliable evaluation of the algorithm’s performance. The AI used in this study includes a quality algorithm that screens raw phonocardiogram signals and removes noise and other artifacts, allowing high-quality phonocardiogram segments to be used for analysis of heart sounds.

A prerequisite for the development and utilization of AI in health care is the ability to reliably listen to and record heart sounds. In recent decades, electronic stethoscopes have undergone significant development, resulting in enhanced capabilities for analyzing murmurs. These devices improve sound signals and reduce background noise, facilitating auscultation. Most available models not only aid in listening but also can record sounds and store data on murmurs for future reference [14].

The accuracy of algorithms primarily hinges on the quantity and quality of the heart sound samples used in their development. The training data must encompass a sufficient variety of normal heart sounds, innocent murmurs, and abnormal murmurs associated with different CHDs. AI algorithms trained with large high-quality datasets outperform interpretations made by inexperienced listeners when assessing murmurs. In this study, the AI was trained on a dataset comprising heart sound samples from 1413 patients, after which it was prospectively tested with 98 new cases. Compared to other studies that have used AI in the analysis of heart sounds in children, the algorithm used in our study was trained with a larger number of samples [9, 15].

In our study, electronic stethoscope recording was performed with four standard anterior auscultation points. This technique differs from that used in a small pilot study, in which the recording was made at the loudest location of the murmur [15]. Murmurs caused by some CHDs are best heard in areas not covered by standard auscultation points. For example, a PDA murmur is often heard just below the left clavicle, and small VSD murmurs are only heard in very small areas. A coarctation of aorta (CoA) murmur is usually heard most clearly from the back near the left scapula. In our study, the algorithm identified all CoAs as abnormal even though the back was not included in the recording areas. However, the possibility of a false negative result increases if heart sounds are analyzed in an area where the murmur is not heard best. The ability of an algorithm to identify CHD is also weakened if the murmur associated with it does not clearly differ from innocent murmurs. ASD can occur without a murmur, or the murmur of ASD can mimic an innocent murmur from the pulmonary artery area, which was also observed in this study as a false negative finding for ASD. The ability of AI to recognize murmurs outside standard areas can be improved by directing the recording to the point where the physician hears the murmur best. To improve the reliability of murmur examinations in children, a promising approach would be to combine the results of an AI algorithm with findings from a clinical examination.

AI algorithms based on the recognition of murmurs and normal heart sounds are unable to recognize CHDs without an audible murmur. Therefore, heart defects without murmurs could not be used to train our algorithm and were also excluded from our training dataset. In this study, AI failed to recognize ASDs, small VSDs, and bicuspid aortic valves with normal function (no stenosis or insufficiency) without a murmur. These defects represent “false negatives” and explain the decrease in sensitivity in the entire study population, which also included patients without a murmur. The age group over 4 years had more CHDs without a murmur, which explains the lower sensitivity in the older age group in our study. Some of the critical or duct-dependent CHDs do not cause a murmur at the early phase, and there were only three patients under 1 month of age in our study group. Thus, this AI algorithm cannot be utilized in the screening of neonate’s critical CHDs, but their detection is still based on fetal anomaly screening, pulse oximetry screening, and clinical status.

Breathing sounds and heart rate can affect the quality of heart sound recordings [10]. Both are faster in children than in adults, and both decrease as the child ages. The effect of breathing sounds on the quality of murmur recordings can be mitigated by performing the recordings during breath holding, as reported in a small pilot study [15]. However, breath holding requires good co-operation and is not possible for small children. Algorithms based on adult heart sound samples cannot be used for screening children, as heart diseases and murmurs differ between children and adults. Our algorithm was developed with samples from different age groups of children and adolescents (from 0 to 18 years), so it could be a promising method for broader use in the field of pediatrics and adolescent medicine.

Previous validation studies assessing AI algorithms in the identification of pathologic murmurs in children have reported similar results to ours. In a virtual clinical study (*n* = 120, age 2–17 years) based on a database of recordings of children’s heart sounds and murmurs, AI identified Still’s innocent murmur with sensitivity of 90% and specificity of 98%. This selected patient sample had no other innocent murmurs or normal heart sounds, and only sound samples recorded at the lower left sternal border were used in the analysis, distinguishing it from our study. The performance of the algorithm worsened when also the sound samples without a murmur and all auscultation areas were included, resulting in a sensitivity of 83% and specificity of 89% [16]. A small (*n* = 34) AI pilot study in children over 3.5 years of age reported a sensitivity of 87% and a specificity of 100% in identifying pathologic murmurs [15]. A virtual clinical trial (*n* = 603) using AI identified pathologic murmurs with a sensitivity of 93% and a specificity of 81%. In that study, previously recorded patient heart sounds were analyzed from a sound bank with the help of AI. Pathologic cases had at least one pathologic diagnosis by echocardiogram and at least one murmur considered to be caused by the pathology. CHD patients without a murmur or with innocent murmurs were excluded, which increased the accuracy of the algorithm [10]. However, due to the differences in patient selection, these results cannot be directly compared with those of our study. The virtual clinical trial by Thompson et al. included also adults, and the age range was wide (0.3–80.9 years), with a median age of 8.8 years and 34% of the patients being over 12 years of age [10].

The strength of our clinical study is the use of echocardiography combined with AI analysis and clinical examination. Another strength of our study is that versatile data were collected from different age groups, covering normal heart sounds as well as innocent and pathologic murmurs related to CHDs. In addition, a large dataset of over 1400 sound samples, validated with echocardiography, was used in developing the algorithm.

One of the limitations of this study was the small number of children under 1 month of age with fast heart and respiratory rates, raising questions about the algorithm’s utility in that age group, which warrants further evaluation. The intensity grades of the murmurs or the severity levels of the heart defects were not included in the research data and therefore could not be used in this study. The effects of heart rates and interval times affecting the output of the algorithm were not studied and excluded from the research, but it is an interesting question indeed. In addition, the exclusion of children with prior heart surgeries means it was not possible to assess the algorithm’s effectiveness in identifying murmurs in this specific pediatric population. The inclusion of children with innocent murmurs makes it difficult to compare the results to those of previous studies [10, 15]. The algorithm is not able to give a specific diagnosis of a heart defect or distinguish the degree of severity of heart defects. The goal of the developed algorithm is not to diagnose a specific heart defect but to serve as a screening method for distinguishing between innocent and pathological murmurs. The purpose of this study was to investigate the suitability of the screening method in the patients of the cardiac outpatient clinic.

In Finland, most referrals due to murmurs or suspicion of CHD come from primary health care, so our algorithm could be most useful in screening for murmurs in that context [7]. The high prevalence of innocent murmurs detected in primary health care strains the limited resources of specialized care. Evaluations of auscultatory findings by inexperienced listeners leads to increased numbers of referrals to specialized medical services. Thus, if innocent murmurs could be reliably diagnosed in primary care settings using AI as an aid to clinical examination, the costs of specialized care could be reduced. Then, direct specialized health care resources could be targeted to those patients who need them most.

In conclusion, the AI algorithm developed in this study showed promising results among pediatric cardiology outpatients in distinguishing between innocent and pathologic murmurs, exhibiting good sensitivity and specificity. It could be used as an aid to identify murmurs that require further analysis by echocardiography. In addition, when combined with clinical examination, the use of this AI algorithm could increase the number of accurate diagnoses of benign murmurs without a need for echocardiography, thus decreasing health care expenses. Additional research is needed to investigate the potential application of AI algorithms in primary health care settings for screening murmurs in children. A working algorithm could be most useful in developing countries, in which the availability of echocardiography can be limited [17].

## Data Availability

No datasets were generated or analysed during the current study.

## Abbreviations

AI: Artificial intelligence
ASD: Atrial septal defect
CHD: Congenital heart defect
CI: Confidence interval
CoA: Coarctation of aorta
HUS: Helsinki University Hospital
PDA: Patent ductus arteriosus
PS: Pulmonary stenosis
VSD: Ventricular septal defect
